# ADP-Mediated Upregulation of Expression of CD62P on Human Platelets Is Critically Dependent on Co-Activation of P2Y1 and P2Y12 Receptors

**DOI:** 10.3390/ph13120420

**Published:** 2020-11-24

**Authors:** Ronald Anderson, Annette J. Theron, Helen C. Steel, Jan G. Nel, Gregory R. Tintinger

**Affiliations:** 1 Department of Immunology, Faculty of Health Sciences, University of Pretoria, Pretoria 0001, South Africa; atheron@uplac.za (A.J.T.); helen.steel@up.ac.za (H.C.S.); 2 Department of Haematology, Faculty of Health Sciences, University of Pretoria, Pretoria 0001, South Africa; Jan.nel@up.ac.za; 3 Tshwane Academic Division of the National Laboratory Health Service of South Africa, Pretoria 0001, South Africa; 4 Department of Internal Medicine, Faculty of Health Sciences, University of Pretoria, Pretoria 0001, South Africa; gregory.tintinger@up.ac.za

**Keywords:** adenosine 5′-diphosphate, cytosolic calcium, phospholipase C, phosphatidylinositol 3-kinase, platelets, P2Y1/P2Y12 receptors

## Abstract

This study probed the differential utilization of P2Y1 and P2Y12 receptors in mobilizing CD62P (P-selectin) from intracellular granules following activation of human platelets with adenosine 5′-diphosphate (ADP, 100 µmol·L^−1^) Platelet-rich plasma (PRP) was prepared from the blood of adult humans. CD62P was measured by flow cytometry following activation of PRP with ADP in the absence and presence of the selective antagonists of P2Y1 and P2Y12 receptors, MRS2500 and PSB0739 (both 0.155–10 µmol·L^−1^), respectively. Effects of the test agents on ADP-activated, CD62P-dependent formation of neutrophil:platelet (NP) aggregates were also measured by flow cytometry, while phosphatidylinositol 3-kinase (PI3K) activity was measured according to Akt1 phosphorylation in platelet lysates. Treatment with MRS2500 or PSB0739 at 10 µmol·L^−1^ almost completely attenuated (94.6% and 86% inhibition, respectively) ADP-activated expression of CD62P and also inhibited NP aggregate formation. To probe the mechanisms involved in P2Y1/P2Y12 receptor-mediated expression of CD62P, PRP was pre-treated with U73122 (phospholipase C (PLC) inhibitor), 2-aminoethoxy-diphenyl borate (2-APB, inositol triphosphate receptor antagonist), calmidazolium chloride (calmodulin inhibitor), or wortmannin (PI3K inhibitor). U73122, 2-APB, and wortmannin caused almost complete inhibition of ADP-activated expression of CD62P, while calmidazolium chloride caused statistically significant, partial inhibition. PSB0739, but not MRS2500, caused potent inhibition of PI3K-mediated phosphorylation of Akt1. Optimal mobilization of CD62P by ADP-stimulated platelets is critically dependent on the co-activation of platelet P2Y1 and P2Y12 receptors. P2Y12 receptor activation is the key event in activation of PI3K, while activation of the P2Y1 receptor appears to create a high cytosolic Ca^2+^ environment conducive to optimum PI3K activity.

## 1. Introduction

Platelets are well recognized as being key players in orchestrating inflammatory responses, particularly those involving neutrophils, driving activities such as heterotypic neutrophil:platelet (NP) aggregation, adhesion to vascular endothelium, extravasation, and formation of neutrophil extracellular traps (NETs) [1,2,3,4,5,6,7]. In this setting, mobilization of the adhesion molecule, CD62P (P-selectin), located in platelet α-granules, is a major trigger of NP interactions via binding to its counter ligand, P-selectin glycoprotein ligand-1 (PSGL-1), constitutively expressed on neutrophils and other types of leukocyte [4,5,7]. Although platelet activation per se, as well as platelet-driven neutrophil activation, are potentially protective against microbial and viral pathogens [8,9,10], if excessive, these events increase the potential risk of intravascular occlusion [1,7,11,12,13,14]. This contention is supported by findings that NP aggregates, NETs, as well as NET-derived constituents, are major components of intravascular thrombi [11,12,13,14].

Although various physiologic and non-physiologic activators of platelets have been described, the most effective primary, biological activators are firstly, thrombin in particular, generated by tissue factor activation, and, secondly, sub-endothelial-exposed collagen, which promote activation of the intrinsic and extrinsic coagulation pathways respectively. Thrombin initiates platelet activation via engagement of the type 1 proteinase-activated receptor (PAR1) and, albeit to a lesser extent, PAR4, while collagen-mediated activation of these cells results from interaction with the glycoprotein (GP) VI receptor [15,16,17]. Common to activation of platelets by thrombin and collagen is the triggering of secondary, autocrine mechanisms of activation, which intensify pro-thrombotic activity. The most important of these are the release of adenosine 5′-diphosphate (ADP) stored in platelet dense granules, as well as thromboxane A_2_ (TXA_2_) generated via the cleavage of membrane phospholipids by phospholipase A_2_ and the sequential actions of cyclooxygenase 1 and thromboxane synthase. TXA_2_ interacts with the prostanoid TP receptor, while ADP activates two major platelet-activating purinergic receptors viz. P2Y1 and P2Y12 [16], functioning both as a primary activator of platelets as well as a cofactor, augmenting responses to other primary activators.

P2Y1 and P2Y12 receptors are diverse types of G-protein-coupled receptors, which are believed to harmonize in activating ADP-mediated platelet aggregation [18,19], with the P2Y1 receptor having a lower receptor density of around 150/cell compared to an estimated 425/cell in the case of P2Y12 [20]. However, the specifics of the mechanisms underpinning the cooperative interactions between platelet P2Y1 and P2Y12 receptors in driving ADP-mediated upregulation of expression of CD62P in particular and resultant augmentation of the pro-inflammatory responses of leukocytes are somewhat less clear [10,21,22,23,24,25]. 

Accordingly, in the current study, we investigated the relative contributions of P2Y1 and P2Y12 receptors to the mobilization of CD62P during activation of platelets with ADP, as well as mechanisms involved in intracellular signalling. The experimental approach involved measurement of expression of CD62P and formation of NP aggregates by ADP-activated platelets in the absence and presence of MRS2500 and PSB0739, which are highly potent, selective inhibitors of platelet P2Y1 and P2Y12 receptors, respectively [26,27]. We probed mechanisms involved in receptor-mediated, cooperative intracellular signalling using a range of pharmacological antagonists of signal transduction. In addition, the effects of MRS2500 and PSB0739 on phosphatidylinositol 3-kinase (PI3K)-mediated phosphorylation of platelet Akt1, which is the predominant isoform of this serine/threonine protein kinase in platelets [28] and a key event in platelet activation [29], were also investigated.

## 2. Results

### 2.1. Purified Platelet Counts

The mean number of platelets in the purified suspensions (*n* = 6) was 243 ± 166 × 10^9^ L^−1^.

### 2.2. Effects of MRS2500 and PSB0739 on Expression of CD62P by ADP-Activated Platelets

**Figure 1:**
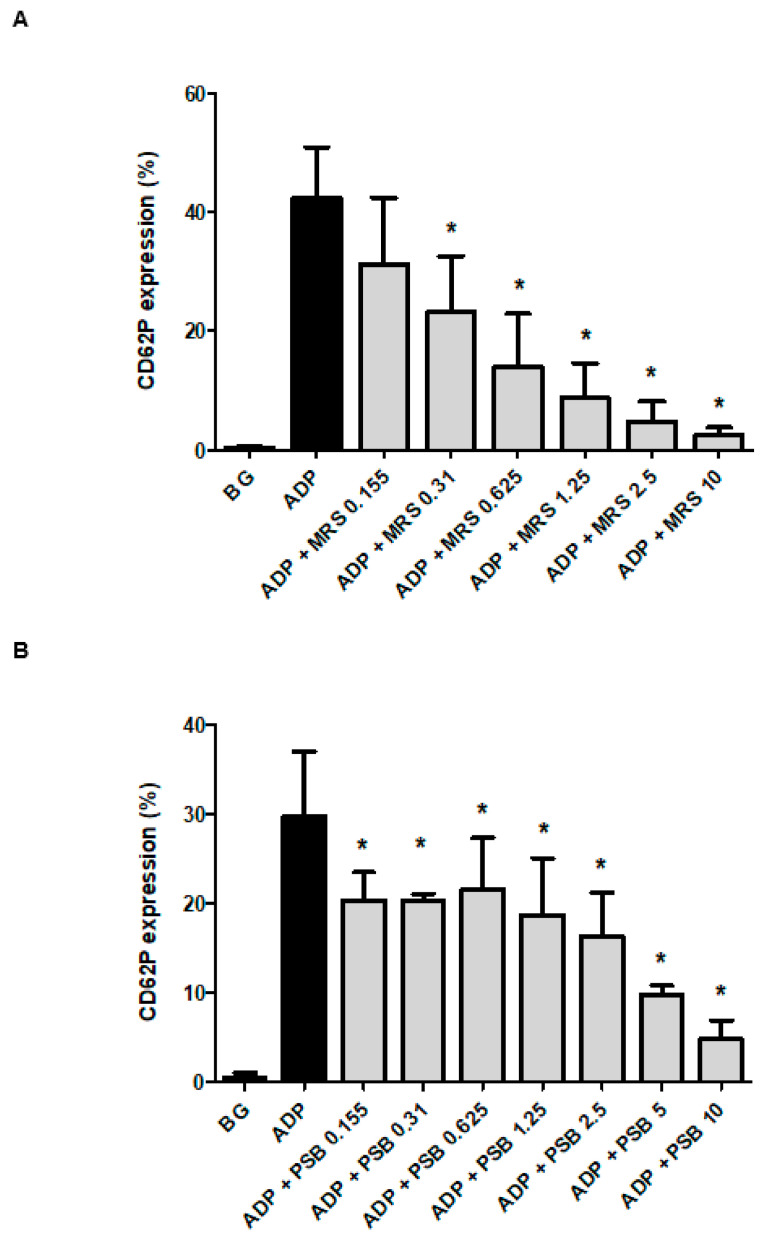
Effects of MRS2500 (MRS) (**A**) and PSB0739 (PSB) (**B**), (0.155–10 μmol·L^−1^) on the magnitude of expression of CD62P on platelets activated with ADP (100 µmol·L^−1^). The results are expressed as the mean% activated platelets ± SDs. * p < 0.05–*p* < 0.001 for comparison with the drug-free control system. BG = unstimulated platelets (*n* = 4–6 individual donors with 4–8 experiments in each series).

The effects of MRS2500 and PSB0739 (both at 0.155–10 µmol·L^−1^) on the expression of CD62P by ADP-activated platelets, relative to those of drug-free control cells, are shown in Figure 1A,B, respectively. The CD62P expression levels of the drug-free, unstimulated control system (in the absence of ADP) were minimal, increasing significantly following the addition of ADP. Pre-treatment of PRP with either MRS2500 or PSB0739, resulted in statistically significant, dose-related inhibition of ADP-activated expression of CD62P, which was almost complete at a concentration of 10 µmol·L^−1^ of MRS2500 and PSB0739 (94.6% and 86.0% inhibition, respectively; *p <* 0.001).

The inhibitory effects of MRS2500 at concentrations of 0.155 and 0.31 µmol·L^−1^ and PSB0739 at 2.5 µmol·L^−1^, individually and in combination, are shown in Table 1. The respective mean levels of inhibition observed with the individual agents were 29%, 48%, and 37%, while the magnitude of inhibition of expression of CD62P observed with the combination of MRS2500 at 0.155 and PSB0739 at 2.5 µmol·L^−1^ was 60%. The corresponding level of inhibition observed with the combination of 0.31 µmol·L^−1^ MRS2500 and 2.5 µmol·L^−1^ PSB0739 was 72%, indicative of a modest augmentative interaction (*n* = 4 individual donors with seven experiments in the series).

### 2.3. Effects of MRS2500 and PSB0739 on Expression of CD62P by Thrombin- and U46619-Activated Platelets

**Figure 2:**
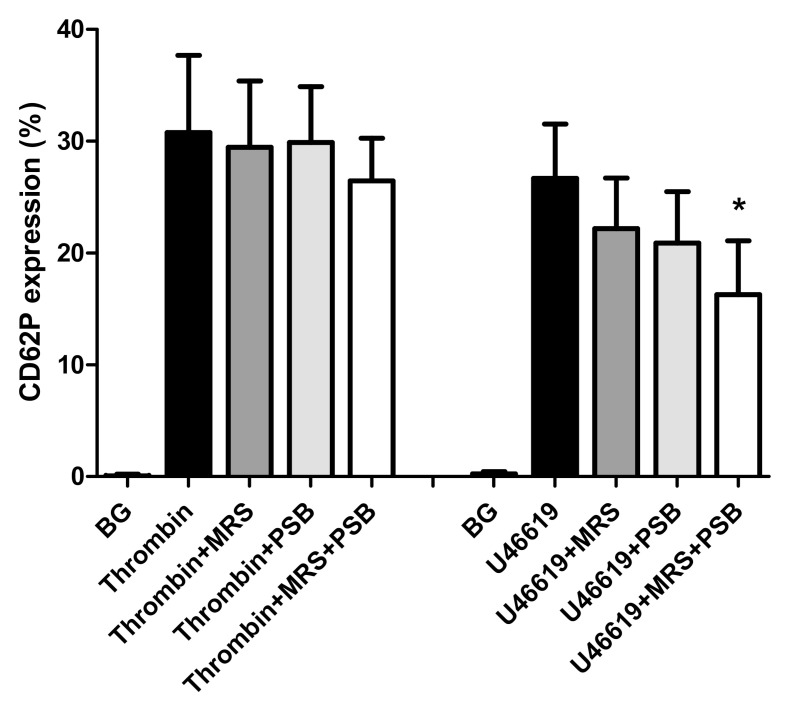
Effects of MRS2500 (MRS) and PSB0739 (PSB) both at 10 µmol·L^−1^ individually and in combination on the magnitude of expression of CD62P on platelets activated with thrombin (0.6 NIH units·mL^−1^) or with U46619 (1 µmol·L^−1^). The results are expressed as the mean% activated platelets ± SDs. * *p* < 0.004 for comparison with the drug-free control system. BG = unstimulated platelets (*n* = 5 individual donors with 7–8 experiments in the series).

These results are shown in Figure 2. Activation of platelets with thrombin resulted in significant upregulation of expression of CD62P, which was not affected by pre-treatment of the cells with either MRS2500 or PSB0739 individually (10 µmol·L^−1^), although slight, statistically non-significant inhibition (14%), was noted in the case of the combination of receptor antagonists. In the case of platelets activated with U46619, pre-treatment of the cells with MRS2500 and PSB0739 individually and in combination resulted in 16.8% (*p* < 0.1), 21.6% (*p* < 0.053), and 39% (*p* < 0.004) inhibition, respectively. The observed lack of effect of MRS2500 and PSB0739 individually on expression of CD62P by thrombin-activated platelets revealed in this series of experiments appears to exclude cytotoxic effects of these agents on platelets. Moreover, the decreased expression of CD62P detected following the activation of MRS2500- or PSB0739-treated platelets with U46619, individually and especially in combination, as well as thrombin-activated cells in the presence of both agents, is seemingly consistent with attenuation of secondary autocrine stimulation due to release of endogenous ADP.

### 2.4. Effects of 2-APB, Calmidazolium Chloride, U73122 and Wortmannin on Expression of CD62P by ADP-Activated Platelets

**Figure 3:**
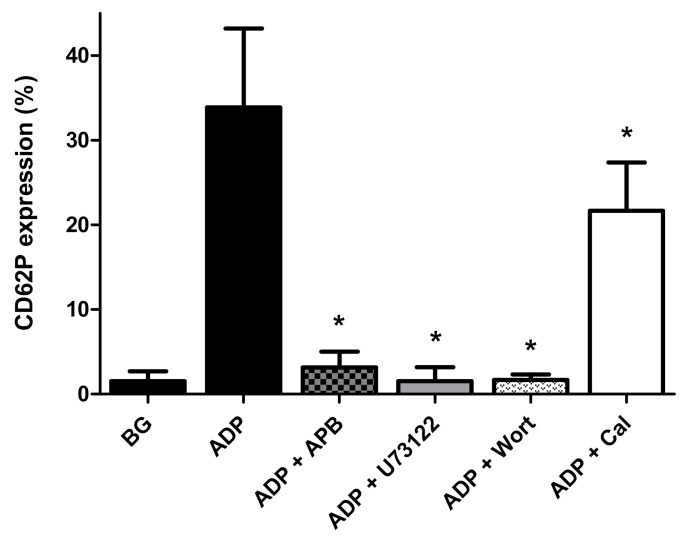
Effects of 2-APB (25 μmol·L^−1^), U73122 (0.625 μmol·L^−1^), wortmannin (Wort, 1 µmol·L^−1^) and calmidazolium chloride (Cal, 10 µmol·L^−1^) on the magnitude of expression of CD62P on platelets activated with ADP (100 µmol·L^−1^). The results are expressed as the mean% activated platelets ± SDs. * p < 0.001–*p* < 0.0002 for comparison with the drug-free control system. BG = unstimulated platelets (*n* = 4 individual donors with 6–18 experiments in each series).

These results are shown in Figure 3. All four antagonists of signal transduction caused statistically significant inhibition of expression of CD62P by ADP-activated PRP that was almost complete in the presence of U73122 (99.5%; *p* < 0.0002) wortmannin (99.5% inhibition; *p* < 0.003) and 2-APB (99% inhibition; *p* < 0.0008), with a mean value of 36% inhibition for calmidazolium chloride (*p* < 0.001). These results are seemingly consistent with the involvement of PLC, PI3K, and calmodulin in ADP-activated expression of CD62P by platelets.

### 2.5. Neutrophil:Platelet (NP) Aggregation

**Figure 4:**
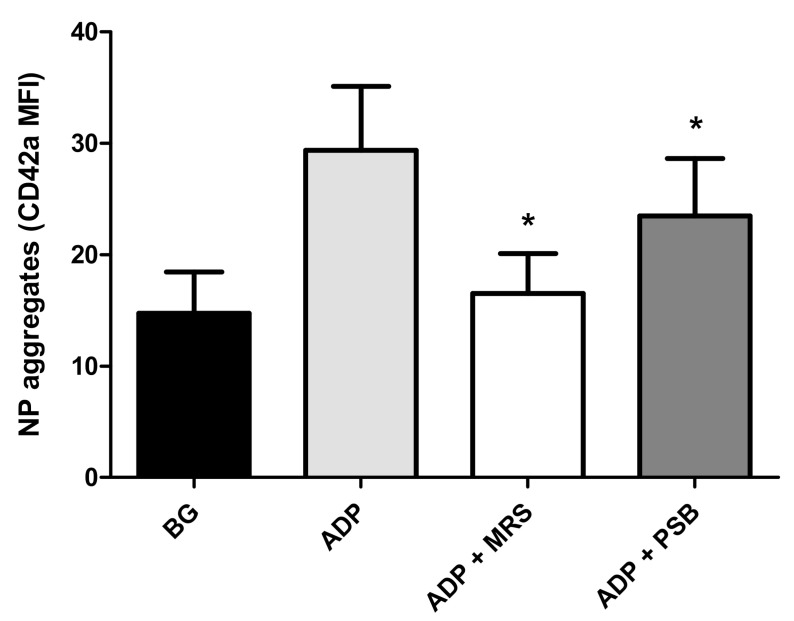
Effects of MRS2500 (MRS) and PSB0739 (PSB) both at 10 µmol·L^−1^ on the formation of NP heterotypic aggregates following addition of ADP (100 µmol·L^−1^). The results are expressed as the mean fluorescence intensity (MFI) of CD42a-PE emitted by CD16^+^/CD45^+^ neutrophils. * p < 0.05–*p* < 0.0002 for comparison with the drug-free control system. BG = unstimulated platelets (*n* = 5 individual donors with 9–17 experiments in the series).

The effects of MRS2500 and PSB0739 (both at 10 µmol·L^−1^) on the formation of NP heterotypic aggregates following the addition of ADP (100 µmol·L^−1^) to buffy coat preparations are shown in Figure 4. Relative to the drug-free, ADP-activated control system, treatment with MRS2500 resulted in almost complete attenuation of ADP-activated formation of NP aggregates (99.7% inhibition; *p* < 0.0002), while lesser effects (42% inhibition; *p* < 0.05) were observed with PSB0739. The greater inhibitory potency of MRS2500 relative to that of PSB0739 with respect to NP aggregate formation may relate to the stronger effects of this agent on platelet CD62P expression.

### 2.6. Effects of MRS2500 and PSB0739 on Akt1 Phosphorylation

**Figure 5:**
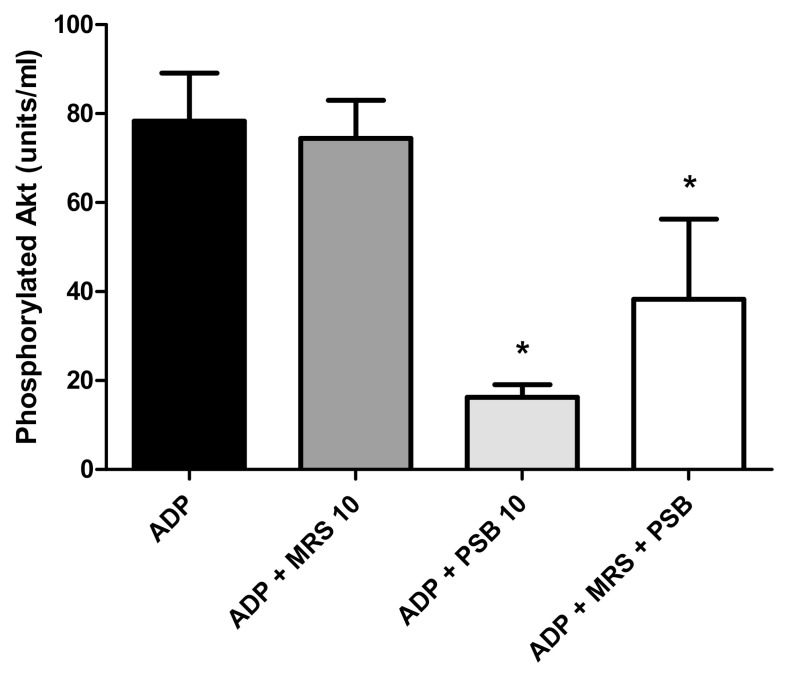
Effects of MRS2500 (MRS) and PSB0739 (PSB) both at 10 µmol·L^−1^, individually and in combination on PI3K-mediated phosphorylation of Akt1 measured in the lysates of ADP (100 µmol·L^−1^)-activated platelets using an ELISA procedure. The results of 5 experiments (*n* = 3 donors) are presented as the mean values ± SDs as units phosphorylated Akt1·mL^−1^. * *p* < 0.008 for comparison of PSB0739 alone and in combination with MRS 2500 with the control system.

As shown in Figure 5, the pre-treatment of isolated platelets with PSB0739 (10 µmol·L^−1^), but not MRS2500 (10 µmol·L^−1^), resulted in potent, statistically significant (*p* < 0.008) inhibition of PI3K-mediated phosphorylation of Akt1. These effects were also evident when the two agents were used in combination with the inhibitory effects due entirely to PSB0739, representing the probable mechanism by which this agent contributes to attenuation of ADP-induced platelet activation. The lesser inhibitory effects of the combination of PSB0739 with MRS2500 on ADP-activated phosphorylation of Akt1 are somewhat surprising. It is however noteworthy that this is the only series of experiments in which pure platelet suspensions were exposed to the combination of both agents used at 10 µmol·L^−1^, which may have led to non-specific effects.

## 3. Discussion

Our findings demonstrate that the mobilization of CD62P from α-granules following exposure of human platelets to ADP in vitro is markedly attenuated by blockade of either P2Y1 or P2Y12 receptors, consistent with a critical requirement for receptor co-activation. Similar findings were observed for ADP-activated formation of pro-thrombotic/pro-inflammatory NP aggregates, an event in which CD62P plays a key role [4,5,7]. These observations coincide in part with those of several earlier studies, albeit focused on the effects of different types and combinations of purinergic receptor antagonists on platelet activities other than expression of CD62P. In this context, Jin et al. reported that co-activation of P2Y1 and P2T_AC_ (later identified as P2Y12) receptors was essential for platelet aggregation, although CD62P expression and NP aggregate formation were not investigated [30]. Nylander et al. reported similar findings for ADP-mediated activation of the platelet integrin, αIIb/β3 [31].

In the case of CD62P expression, early studies by Storey et al., which were focused solely on P2Y12, reported that pharmacologic blockade of this receptor resulted in marked suppression of both upregulation of expression of CD62P by ADP-activated platelets, as well as formation of platelet:leukocyte aggregates [21,22]. In an extension of these studies, Leon et al. reported that pharmacologic blockade of either P2Y1 or P2Y12 receptors resulted in almost complete inhibition of upregulation of CD62P expression by ADP-activated human platelets [23], as noted in the current study. In contrast to the current study, however, complete attenuation of ADP-activated NP aggregate formation required simultaneous blockade of both receptor types [23]. More recently, Oestreich et al. used MRS2179 and cangrelor to achieve the pharmacologic blockade of platelet P2Y1 and P2Y12 receptors, respectively. These authors observed that antagonism of P2Y1 receptors caused partial inhibition of CD62P expression by ADP-activated human blood platelets, but only at low concentrations of the agonist (<6 µmol·L^−1^), while antagonism of P2Y12 receptors, although more effective, also resulted in incomplete attenuation, which was not augmented by inclusion of a P2Y1 receptor antagonist [24]. In this context, it is noteworthy that MRS2179 has a lower affinity for the P2Y1 receptor than MRS2500, with reported Ki values of 72.5 ± 15 nM and 1.20 ± 0.15 nM, respectively [32].

Although we agree that similarities exist between the findings of the current and earlier studies, there are also significant differences. Importantly, as alluded to above, these may relate to the type of purinergic receptor antagonists used in the various studies, making it difficult to distinguish between receptor synergy, interplay, or strict inter-dependence. In the context of the current study, it is noteworthy that receptor utilization studies were undertaken using a combination of potent, high-affinity, selective antagonists of platelet P2Y1 and P2Y12 receptors, underscoring the veracity of our findings. Unlike most previous studies, we also attempted to exclude lack of receptor selectivity and possible cytotoxicity of MRS2500 and PSB0739. Both scenarios were supported by observations that these agents individually (at 10 µmol·L^−1^) did not significantly affect the expression of CD62P by platelets activated with thrombin. In combination, however, the purinergic receptor antagonists caused modest, albeit statistically insignificant, inhibition of thrombin-activated expression of CD62P, probably due to attenuation of secondary, autocrine activation following mobilization of ADP from platelet α-granules. Of note were the more striking inhibitory effects of MRS2500 and PSB0739 individually, and especially in combination, on the expression of CD62P by platelets activated with the thromboxane A_2_ mimic, U46619. This finding is indicative of a more prominent role played by secondary, ADP-mediated autocrine activation of CD62P expression in platelets activated by thromboxane A_2,_ as opposed to thrombin.

As a strategy to identify mechanisms that may contribute to P2Y1/P2Y12 receptor-mediated harmonization of platelet activation, specifically in relation to the upregulation of expression of CD62P, an additional series of experiments was undertaken using a range of pharmacological inhibitors of intracellular signalling pathways. In this context, intracellular signalling mechanisms activated following engagement of P2Y1 receptors involve the Gαq-protein signal transduction pathway linked to activation of the β2 and β3 isoforms of PLC, which, via the formation of IP_3_, promote the release of Ca^2+^, a key second messenger, from intracellular stores [33]. As reported in the current study, and in agreement with the findings of others [34], treatment of platelets with U73122 almost completely attenuated Ca^2+^ mobilization and upregulation of expression of CD62P. Treatment of the cells with 2-APB, an antagonist of IP_3_ receptors present on intracellular Ca^2+^ stores, also resulted in almost complete inhibition of CD62P expression. Similar, albeit less striking effects, were observed with the calmodulin inhibitor, calmidazolium chloride, possibly via inhibition of calmodulin-dependent coupling of IP_3_ receptors to Ca^2+^ release channels [35,36]. These observations clearly underscore the critical nature of the intensity of the Ca^2+^ signal in mobilization of platelet α-granules.

In addition, elevations in the concentration of cytosolic Ca^2+^ following engagement of platelet P2Y1 receptors by ADP may be augmented by an auxiliary dual mechanism of mobilization of the cation triggered by the G_αi2_ subunit of the P2Y12 G-protein-coupled receptor. These mechanisms involve: (i) inhibition of adenylyl cyclase, which prevents re-sequestration of cytosolic Ca^2+^; and (ii) activation of the β- and γ-isoforms of PI3K [10,30,37,38], which enhance Ca^2+^ signalling via activation of the PLCγ2 isoform [39,40]. Activation of a high intensity Ca^2+^ signal may therefore be a crucial outcome of P2Y1/P2Y12 receptor co-activation and is possibly a prerequisite for optimal mobilization of platelet α-granules. These observations, taken together with the potent inhibitory effects of wortmannin on platelet activation described here and elsewhere [38], are seemingly consistent with a mechanistic interplay between PLC and PI3K in the mobilization of Ca^2+^ by ADP-activated platelets, as reported previously by Hardy et al. [37]. In this context, intracellular Ca^2+^ elevations are apparently necessary to sustain the PI3K activity [41].

However, further exploration of this relationship in the current study has revealed a seemingly additional level of complexity surrounding the events involved in the interplay between the various isoforms of PLC and PI3K and expression of CD62P.

Notwithstanding activation of the β- and γ-isoforms of PI3K following engagement of platelet P2Y12 receptors, several lines of evidence have implicated P2Y1 receptors in the activation of a third isoform of PI3K present in platelets, namely the α-isoform of PI3K. In this context, Ca^2+^/calmodulin, which interacts with both the P2Y1 receptor and PLCβ [42,43], has been reported to activate the α-isoform of PI3K via binding to the SH2 domains of the p85 kDa regulatory subunit of the enzyme [44,45]. PI3Kα, in turn, synergises with PI3Kβ to augment activation of PLCγ2 and Ca^2+^ signalling [46]. Moreover, a link between P2Y1 receptor activation and stimulation of the PI3K/Akt pathway has also been demonstrated in glial cells [47,48]. These findings, together with those of the current study, suggest that the dependence of maximal expression of CD62P, and possibly other pro-inflammatory/pro-thrombotic activities of ADP-activated platelets, on co-activation of P2Y1 and P2Y12 receptors may actually result from cooperative signalling mediated by several isoforms of PLC and PI3K.

**Figure 6:**
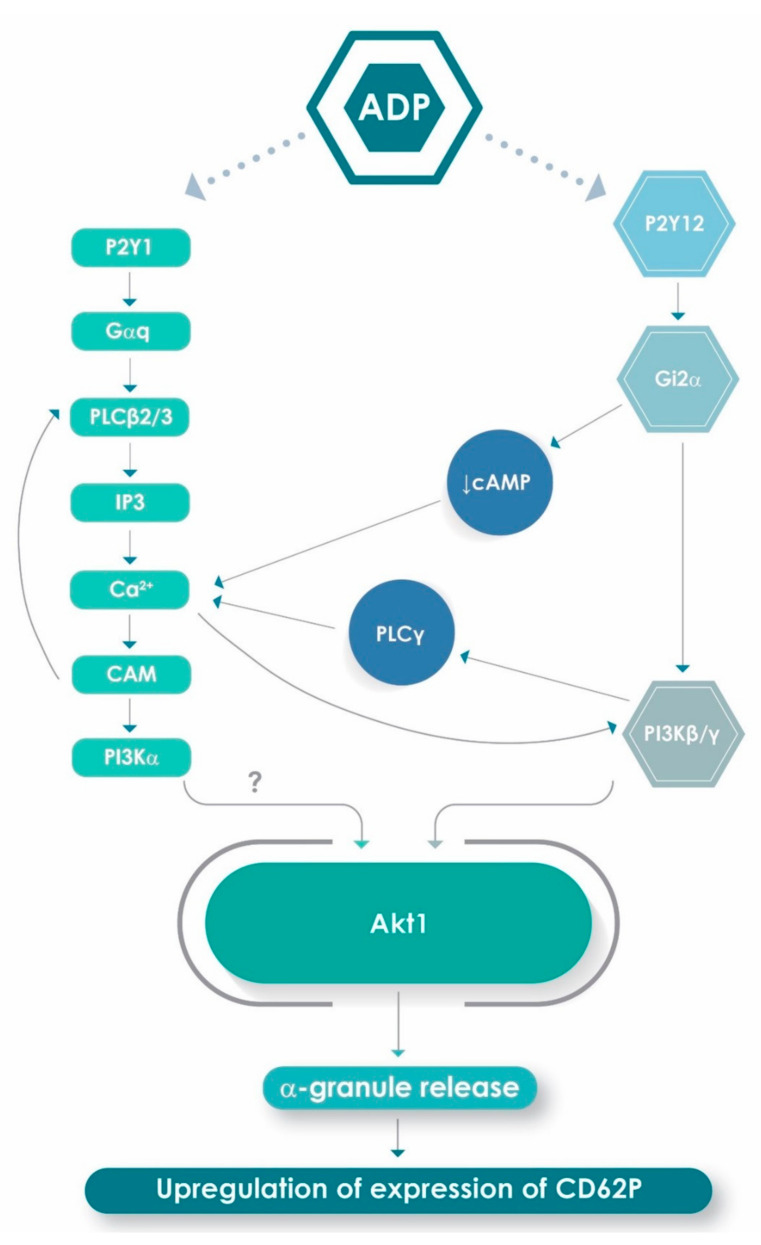
Proposed mechanism by which P2Y1 and P2Y12 platelet purinergic receptors harmonize to induce platelet α-granule release and upregulation of expression of CD62P. Interaction of ADP with P2Y1 Gαq protein-coupled receptors and P2Y12 Gi2α protein-coupled receptors leads to triggering of an initially divergent series of intracellular signalling events. In the case of P2Y1, ligation of ADP results in activation of phospholipases C (PLC) β2 and β3, mobilization of intracellular Ca^2+^ and possibly calmodulin (CAM)-mediated activation of the α-isoform of phosphatidylinositol 3-kinase (PI3K, indicated by ?). This series of intracellular, Ca^2+^-dependent signalling events is augmented via activation of P2Y12 receptors due to Gi2α protein-mediated: (i) inhibition of adenylyl cyclase, preventing re-sequestration of cytosolic Ca^2+^; and (ii) recruitment of the β- and γ-isoforms of PI3K, which, in turn, activate the γ-isoform of PLC. Activation of PLCγ is further augmented via PI3Kβ/γ interactions, augmenting the high cytosolic Ca^2+^ milieu seemingly necessary for optimal activity of PI3K. Finally, the activated isoforms of PI3K harmonize to promote activation of Akt1, a seemingly critical event in mobilization of platelet α-granules and upregulation of expression of CD62P.

To probe the direct involvement of the P2Y1 and P2Y12 receptors in the activation of PI3K, we investigated the effects of treatment of isolated human platelets with MRS2500 and PSB0739 individually and in combination on the PI3K-mediated phosphorylation of Akt1, a key event in ADP-activated α-granule mobilization [29]. These experiments revealed that the pre-treatment of ADP-activated platelets with PSB0739, but not MRS2500, resulted in potent inhibition of phosphorylation of Akt1. These findings, taken together with those of the aforementioned studies, using the various pharmacological inhibitors of intracellular signalling mechanisms, appear to support the contention that co-activation of P2Y1 and P2Y12 receptors results in cooperative signalling mediated by several isoforms of PLC and PI3K. This, in turn, underpins optimum expression of CD62P and possibly other pro-inflammatory/pro-thrombotic activities of ADP-activated platelets. A proposed scheme linking P2Y1 and P2Y12 receptor co-activation to driving a high cytosolic Ca^2+^ milieu and activation of PI3K that are necessary to achieve optimum ADP-activated platelet α-granule release is presented in Figure 6.

Notwithstanding our use of, albeit well-recognized, pharmacological agents to target intracellular signalling mechanisms, which may be considered as a potential limitation in the absence of structurally inactive mimics, the sole focus of our study on regulation of purinergic receptor-mediated activation of expression of CD62P could also be interpreted as an additional limitation. We believe, however, that this latter point represents a strength. This contention is based on the increasing recognition of the role played by platelets in orchestrating potentially harmful inflammatory responses, particularly those involving neutrophils, in which CD62P is prominent [4,5,7,49]. Consequently, P2Y1 and P2Y12 receptors are recognized as being attractive targets for anti-thrombotic/anti-inflammatory therapy, which has been achieved in the case of the latter [50]. In the clinical setting, however, optimal attenuation of ADP- and possibly thromboxane A_2_-mediated autocrine activation of platelets may require dual blockade of both P2Y1 and P2Y12 receptors. Nevertheless, co-receptor blockade in the clinical setting must be viewed in the context of the potential risk of bleeding complications. Furthermore, the predominant role played by platelet P2Y1 receptors in certain types of allergic inflammation [10] also underscores the requirement for clinically effective, selective pharmacological antagonists of this type of purinergic receptor.

On the other hand, the apparent minor involvement of ADP-mediated autocrine triggering of the upregulation of expression of CD62P by thrombin-activated platelets confirms the existence of differential mechanisms of CD62P expression in platelets activated with ADP or thrombin. Given this scenario, optimal pharmacologic control of harmful CD62P-orchestrated platelet pro-thrombotic/pro-inflammatory responses in the therapeutic setting may therefore require the addition of the thrombin PAR-1 antagonist, vorapaxar [51], to standard dual anti-platelet therapy, albeit with an increased risk of bleeding.

## 4. Materials and Methods

### 4.1. Ethical Statement

Permission to undertake this study, and to draw blood from healthy, adult human volunteers (*n* = 39; mean age 37.64 ± 15 years; 31 females; 8 males) was granted by the Research Ethics Committee of the Faculty of Health Sciences, University of Pretoria in full compliance with the World Medical Association Declaration of Helsinki 2013 (Approval No. 116/2017). The current study is a sub-study of a comprehensive investigation focused on pharmacological modulation of platelet and neutrophil function. We obtained prior written informed consent from all blood donors, each of whom underwent a routine health check (including measurement of blood pressure) by an experienced, qualified nurse before the blood was drawn.

### 4.2. Chemicals

MRS2500 {(1R*, 2S*)-4-[2iodo-6(methylamino)-9*H*-purin-9-yl]-2-(phosphonoxy)bicyclo[3.1.0]hexane-1-methanol dihydrogen phosphate ester tetraammonium salt; selective inhibitor of P2Y1 receptors}; PSB0739 {1-amino-9,10-dihydro-9,10-dioxo-4[[-(phenylamino)-3-sulf,ophenyl]amino]-2-anthracenesulphonic acid sodium salt; selective antagonist of P2Y12 receptors}; and U73122 {1-[6[[(17β)-3-methoxyestra-1,3,5(10)-trien-17-yl]amino]hexyl]-1*H*-pyrrole-2,5-dione; phospholipase C (PLC) inhibitor} were purchased from Tocris Bioscience (Bristol, UK). The remaining chemicals were purchased from Sigma-Aldrich (St Louis, MO, USA). These were: calmidazolium chloride{1-bis(4chlorophenyl)methyl-3-[2,4-dichloro-β-(2;4-dichlorobenzyloxy)phenethyl]imidazolium chloride; calmodulin inhibitor}, 2-aminoethoxydiphenyl borate [2-APB; antagonist of inositol triphosphate (IP_3_) receptors] and wortmannin {[(1R, 3R, 5S, 9R, 18S]-18-(methoxymethyl)-1-5-dimethyl-6,11,16-trioxo-13,17-dioxapentacyclo[10.6.1.0^2,10^.0^5,9^.0^15,19^]nondeca-2(10),12(19),14-trien-3-yl]acetate; inhibitor of PI3K}. With the exceptions of MRS2500 and PSB0739 (both water-soluble), all of the other agents were dissolved in dimethylsulfoxide (DMSO) with appropriate solvent controls included for each series of experiments.

In the various assays of ADP-activated expression of CD62P by platelets and/or NP heterotypic aggregate formation, the aforementioned agents were used at final, pre-determined concentrations of 0.155–10 µmol·L^−1^ in the case of MRS2500 and PSB0739, while calmidozolium chloride, U73122, 2-APB and wortmannin were used at 10 µmol·L^−1^, 0.625 µmol·L^−1^, 25 µmol·L^−1^ and 0.5 µmol·L^−1^, respectively. In all experiments, platelet-rich plasma (PRP) or buffy coat were pre-incubated with the various test pharmacological agents for 10 min at 37 °C prior to the addition of ADP, followed 5 min later by flow cytometric analysis of platelet CD62P expression and NP aggregate formation as described below. In the case of calmidazolium chloride, 10 µmol·L^−1^ was the maximum concentration that could be used due to apparent inhibition of the plasma membrane Ca^2+^-ATPase at higher concentrations.

The platelet activators used were, firstly, and most importantly, adenosine-5′-diphosphate, as well as thrombin (from human plasma), purchased from Boehringer Mannheim Biochemical (Basel, Switzerland) and the Sigma Chemical Co., respectively, used at final respective concentrations of 100 µmol·L^−1^ and 0.6 NIH units·mL^−1^. A third activator, namely U46619 (Z)-7-[(1*R*,4*S*,5*S*,6*R*)-6-[(*E*,3*S*)-3-hydroxyoct-1-enyl-2-oxabicyclo[2.2.1]heptan-5yl]hept-5-enoic acid, which is a thromboxane A_2_ surrogate (Tocris Bioscience, Bristol, UK) was used at 1 µmol·L^−1^ (final).

### 4.3. Platelet-Rich Plasma and Buffy Coat Preparation

To prepare PRP, blood (anti-coagulated with five units of preservative-free heparin·mL^−1^ blood) was centrifuged at 250× *g* for 10 min at room temperature within 15 min of venepuncture as described previously [52]. The essentially erythrocyte- and leukocyte-free upper layer of PRP was decanted and used in the experiments described below. Buffy coat suspensions prepared by the sedimentation of heparinized venous blood were used in assays of NP aggregate formation [53].

### 4.4. Purified Platelet Suspensions

PRP was diluted four-fold with phosphate-buffered saline (PBS, 0.15M, pH 6.8) containing ethylene glycol-bis(β-aminoethyl ether)-N-N-N’-N’-tetraacetic acid (EGTA, final concentration 0.6 mM, to minimize spontaneous platelet activation) and centrifuged at 250× *g* for 10 min at 20 °C to deplete residual erythrocytes and leukocytes. The platelet-enriched supernates were then centrifuged at 800× *g* for 20 min and the cell pellets resuspended in PBS and counted using a Sysmex XN 20,000 Automated Haematology Analyzer (Sysmex Corporation, Kobe, Japan).

### 4.5. Measurement of Expression of CD62P by Platelets Activated with ADP

For these studies, PRP (20 µL) in a final volume 1 mL Hanks’ balanced salt solution (HBSS, indicator-free, 1.25 mmol·L^−1^ calcium, pH 7.4) was incubated for 5 min at 37 °C in the absence and presence of MRS2500 or PSB0739 (both 0.155–10 µmol·L^−1^, final) individually or in combination (MRS2500 at 0.155 and 0.31 µmol·L^−1^ with PSB0739 at 2.5 µmol·L^−1^). Appropriate solvent controls were included for each test agent. Following incubation, ADP (100 µmol·L^−1^) was added and the tubes incubated for a further 5 min period at 37 °C, after which flow cytometry was performed to determine the magnitude of expression of CD62P according to the proportion (%) of CD42a^+^ platelets expressing the adhesion molecule. The final concentration of ADP was determined as being that which achieved maximal activation of platelets.

In an additional series of experiments, the following were also investigated: (i) the selectivity of MRS2500 and PSB0739 for their respective purinergic receptors by measuring the effects of these agents on the expression of CD62P by platelets activated with thrombin (0.6 NIH units·mL^−1^) or U46619 (1 µmol·L^−1^); and (ii) the effects of 2-APB (25 µmol·L^−1^), calmidazolium chloride (10 µmol·L^−1^), U73122 (0.625 µmol·L^−1^) and wortmannin (1 µmol·L^−1^) on ADP-activated expression of CD62P.

Following incubation with the various test agents, the platelet suspensions (PRP) were stained with 5 µL each of a murine anti-human platelet CD42a-phycoerythrin (PE)-labelled monoclonal antibody (Becton Dickenson, San Jose, CA, USA) and an anti-human CD62P-fluorescein isothiocyanate (FITC)-labelled monoclonal antibody (Beckman Coulter, Miami, FL, USA) to detect the total and activated platelet populations, respectively. After 15 min of incubation in the dark, the samples were analyzed on a Gallios flow cytometer (Beckman Coulter) and the results expressed as the percentage of activated platelets with 50,000 cells interrogated during each measurement. The gating and analytical strategies used in a typical experiment have been described elsewhere [54].

### 4.6. Neutrophil:Platelet (NP) Aggregate Formation

In this more limited series of experiments, the effects of MRS2500 and PSB0739 (both at 10 µmol·L^−1^) on ADP-activated formation of NP aggregates were investigated. Buffy coat (30 µL), suspended in a final volume of 1 mL HBSS, was processed as above for PRP and NP aggregate formation measured following activation with ADP (100 µmol·L^−1^) as described previously [53]. Buffy coat rather than whole blood was used for these experiments to ensure adequate numbers of neutrophils and platelets in the assay system, as well as to minimize the potential influence of ADP derived from erythrocytes. Following incubation, the cells were stained for 15 min at room temperature in the dark with a cocktail consisting of 5 µL of each of the following, murine, anti-human, fluorochrome-labelled monoclonal antibodies: CD16-PC5 (phycoerythrin-cyanine 5; Beckman Coulter), CD42a-PE (Becton Dickenson) and CD45-Krome Orange (Beckman Coulter) to enable detection of neutrophils, platelets and total leukocytes, respectively. This was followed by an analysis of the cell suspensions at a slow flow rate using the Gallios flow cytometer. NP heterotypic aggregate formation was determined as CD16^+^/CD45^+^ neutrophils co-expressing CD42a. Results are expressed as the relative mean fluorescence intensity (MFI) of CD42a as emitted by CD16/CD45 neutrophils as an index of the magnitude of the interaction of platelets with individual neutrophils using the gating strategy described by previously [53].

### 4.7. Measurement of Akt1 Phosphorylation

Platelets (approximately 1 × 10^8^ mL^−1^) suspended in HBSS were pre-incubated with MRS2500 or PSB0739 (10 µg·mL^−1^) individually or in combination for 10 min at 37 °C followed by addition of ADP (100 µmol·L^−1^). After 3 min of incubation [55,56,57], the reactions were terminated and the platelets lysed by addition of an equal volume of cell extraction buffer supplemented with a 1% protease and phosphatase inhibitor cocktail (all purchased from Invitrogen, ThermoFisher Scientific, Carlsbad, CA, USA) and held on ice for 30 min. Thereafter, the cell extracts were centrifuged at 12,000× *g* for 10 min and the supernatants decanted and stored at −80 °C until analysis of phosphorylated Akt1 using an ultrasensitive solid-phase sandwich ELISA system that detects phosphorylation of serine residue 473 on Akt1 (ThermoFisher Scientific). The results of five experiments using platelets from three separate donors are expressed as units phosphorylated Akt1·mL^−1^.

### 4.8. Expression and Statistical Analysis of Results

The results of each series of experiments are expressed as the mean values ± standard deviations (SDs) with the numbers of different donors (*n*) and replicate experiments clearly indicated. Statistical analysis was performed using WinStat statistical software with levels of statistical significance calculated using the Mann-Whitney U-test for comparison of non-parametric data. A *p*-value of <0.05 was considered significant.

## 5. Conclusions

The findings of this study demonstrate a clear dependence of ADP-activated mobilization of CD62P on the co-activation of platelet P2Y1 and P2Y12. With respect to mechanistic issues, the essential cooperative interactions between the two purinergic receptor types in driving the upregulation of CD62P expression by ADP-activated platelets appeared to converge on the generation of a high-intensity Ca^2+^ signal and activation of PI3K. These events appear to involve various isoforms of PLC and PI3K, as well as calmodulin, all of which represent potential therapeutic targets.

## Figures and Tables

**Table 1:** Effect of MRS2500 (0.155 and 0.31 μmol·L^−1^) and PSB0739 (2.5 μmol·L^−1^) individually and in combination on CD62P expression by ADP-activated platelets, as well as the magnitude of inhibition.

System	% CD62 Expression	% Inhibition
BG	0.61 ± 0.22	
ADP 100 μmol·L^−1^	42.4 ± 8	
MRS 0.155 μmol·L^−1^	30.2 ± 8.7 *	29
MRS 0.31 μmol·L^−1^	22 ± 8.8 *	48
PSB 2.5 μmol·L^−1^	26.5 ± 9.3 *	37
MRS 0.155 μmol·L^−1^ + PSB 2.5 μmol·L^−1^	17 ± 7.4 *	60
MRS 0.31 μmol·L^−1^ + PSB 2.5 μmol·L^−1^	12 ± 6.6 *	72

Results are expressed as the mean ± SD. * *p* < 0.05 for comparison with the untreated ADP control.
